# A Nano-Traditional Chinese Medicine Against Lymphoma That Regulates the Level of Reactive Oxygen Species

**DOI:** 10.3389/fchem.2020.00565

**Published:** 2020-07-14

**Authors:** Qiangqiang Zhao, Jian Li, Bin Wu, Yinghui Shang, Xueyuan Huang, Hang Dong, Haiting Liu, Rong Gui, Xinmin Nie

**Affiliations:** ^1^Department of Blood Transfusion, The Third Xiangya Hospital, Central South University, Changsha, China; ^2^Department of Hematology, The Qinghai Provincial People's Hospital, Xining, China; ^3^Department of Transfusion Medicine, Wuhan Hospital of Traditional Chinese and Western Medicine, Tongji Medical College, Huazhong University of Science and Technology, Wuhan, China

**Keywords:** black phosphorus quantum dots, Jolkinolide B, apoptosis, reactive oxygen species, lymphoma

## Abstract

Jolkinolide B (JB) is a bioactive compound isolated from a Chinese herbal medicine that exerts antitumor activity. However, the anti-lymphoma effect of JB and its mechanism are yet to be revealed. Because free JB has poor pharmacokinetics and weak antitumor efficacy, we opted to use black phosphorus quantum dot (BPQD) nanomaterials as a drug loading platform to synthesize a nano-traditional Chinese medicine (nano-TCM) called BPQDs@JB. Compared with free JB, Raji cells administrated with BPQDs@JB exhibited the cell viability of 19.85 ± 1.02%, and the production of intracellular reactive oxygen species (ROS) was promoted. Likewise, BPQDs@JB was capable of rising the apoptosis rate of Raji cells to 34.98 ± 1.76%. In nude mice transplanted tumor model administrated with BPQDs@JB, the tumor tissue sections administrated with BPQDS@JB achieved a conspicuous red fluorescence, demonstrating the presence of most ROS production in the BPQDS@JB. TUNEL achieved a number of positive (brown) nuclei *in vivo*, revealing that BPQDS@JB could significantly induce tumor tissue apoptosis. As revealed from the mentioned results, BPQDs@JB can generate considerable ROS and interfere with the redox state to inhibit tumor. In brief, BPQDs@JB may be adopted as a treatment option for lymphoma.

## Introduction

Jolkinolide B (JB) is a bioactive compound extracted from *Euphorbia fischeriana*, which grows at high altitude and is a traditional Chinese medicine (TCM) with high medicinal value (Yan et al., 2019). In recent years, research on the antitumor and antiviral effects of JB has gained increased attention (Gao et al., 2016; Xu et al., 2016). In fact, previous studies have shown that JB exhibits antitumor effect on numerous tumor cells. For instance, Yan et al. (Gao and Han, 2018) revealed that JB inhibits the proliferation of non-small cell lung cancer cells by downregulating the expression of hexokinase 2. JB can induce apoptosis and anti-metastasis of the breast cancer cell line, MDA-MB-231 (Xu et al., 2013; Sun et al., 2015; Shen et al., 2017). Previously, JB was also found to induce the apoptosis of the human leukemic cells, HL-60 and THP-1 cells, through the JAK2/STAT3 pathway (Wang et al., 2013). The above studies suggest that JB can be used to treat malignant tumors; however, its anti-lymphoma effect has not yet been reported. To provide an experimental basis for its use in the clinic, we aimed to explore the effect of JB on lymphoma and its possible mechanism.

There are some defects in the active ingredient of TCM (e.g., poor water solubility, low bioavailability and rapid clearance *in vivo*), which limits its clinical application to a certain extent (Yang et al., 2011). With the emergence of nanotechnology, however, these problems have been improved. The loading of TCM into nanocarriers can increase their stability and improve their water solubility, bioavailability, and distribution in tumor tissues through the enhanced permeability and retention (EPR) effect on tumors (Khan et al., 2019). Because of its good biocompatibility, high specific surface area, and drug loading rate (Shao et al., 2016), black phosphorus nanoparticle quantum dot (BPQD) serves as an ideal candidate carrier for drug delivery and antitumor therapy (Li et al., 2017).

BPQD, an ultra-small derivative of BP nanosheet, was discovered in 2015 (Zhang et al., 2015). Because P is a key element in the human body, BPQP can be degraded into non-toxic and biocompatible phosphorus oxide (phosphate or phosphonate), which is well tolerated in the human body (Wang et al., 2015). Guo et al. (2018) confirmed that BPQDs do not exhibit evident cytotoxicity and can be cleared by the kidney. Furthermore, Huang et al. (2019) used erythrocyte membrane camouflage BPQDs combined with doxorubicin and Kirenol as an antitumor therapy. Shang et al. (2019) used BPQDs to load Hederagenin to mediate apoptosis and autophagy against breast cancer. Based on the above results, BPQD is a non-toxic, safe, and efficient nano-drug delivery platform.

**Figure 1:**
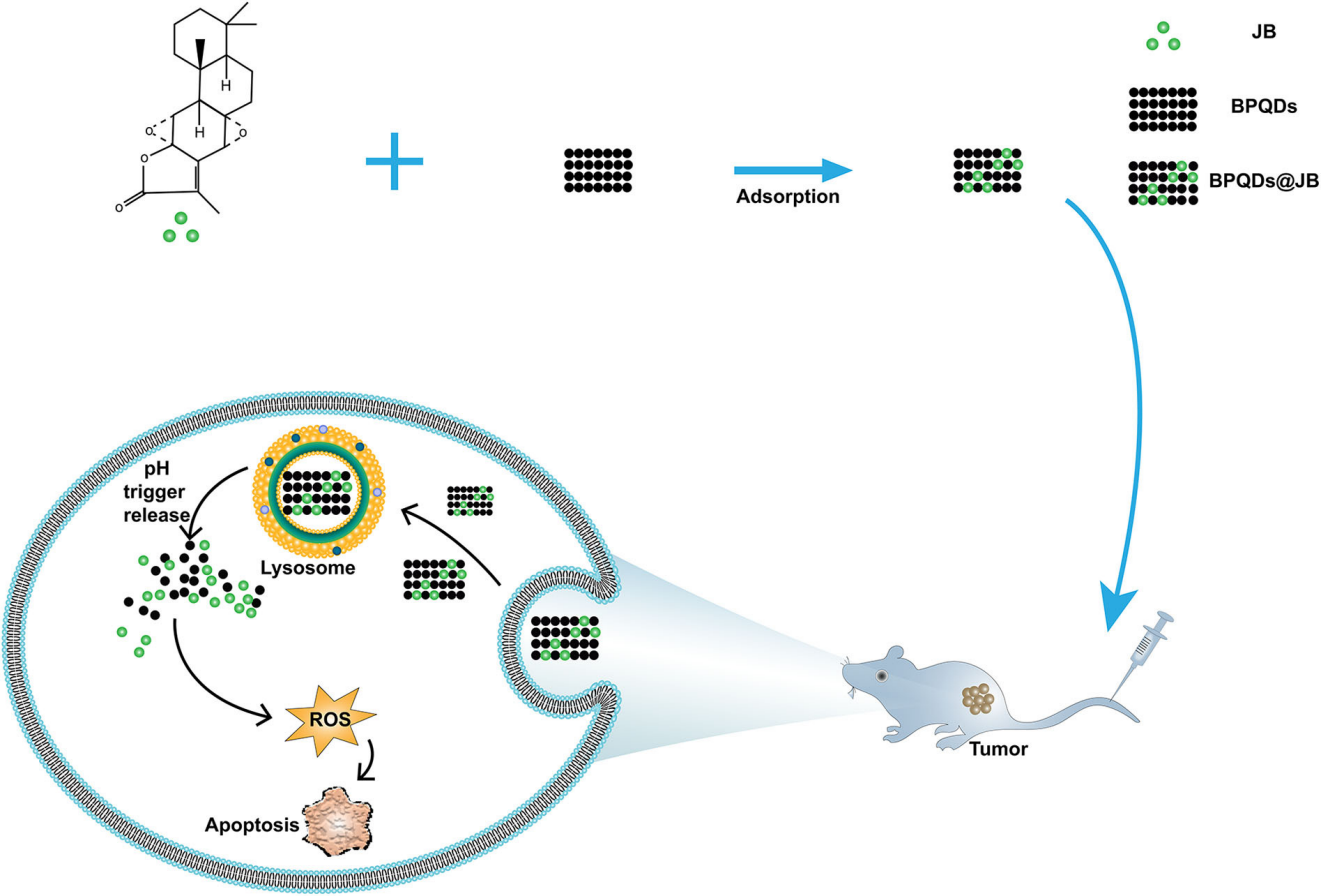
Construction of BPQDs@JB and the schematic diagram of its anti-lymphoma mechanism.

To provide a reference for the clinical treatment of lymphoma with nano-TCM according to the above findings, we aimed to construct and synthesize BPQDs@JB nano-TCM and perform a preliminary assessment to derive the strategy and mechanism of this anti-lymphoma TCM nanodrug delivery system (Figure 1).

## Materials and Methods

### Materials

The BPQD dispersions were purchased from Yuanduo Biotechnology (China). JB was purchased from Desite Biology (China). DMSO and the dialysis membranes (2 kD) were obtained from Solarbio Science & Technology (China). The Annexin V-FITC/PI apoptosis detection kit was purchased from BD Biosciences (China). The ROS test kit was obtained from Beyotime Biotechnology (China). The CCK-8 cell counting kit was purchased from Dojindo Chemical Technology (Japan). Fetal bovine serum (FBS) and RPMI-1,640 were purchased from Biological Industries (Israel). TdT *in situ* apoptosis detection kit was purchased from R&D Systems (China). DAPI was produced by Servicebio Technology (China). The polycarbonate porous membrane syringe filter (200 nm) was purchased from Whatman (USA).

### Cell Culture

Human lymphoma Raji cells were purchased from the Advanced Research Center of Central South University and cultured in RPMI-1,640 medium containing 10% fetal bovine serum. The cells were cultured in a cell incubator at 37°C and 5% CO_2_.

### Preparation of BPQDs@JB

PBS we used was sterilized at high temperature to reduce the solubility of oxygen in water, i.e., oxygen could be released. BPQDs (1 mg) was dissolved in PBS. JB (1 mg) was dissolved in DMSO. Afterwards, BPQDs@JB was built in a nitrogen-filled environment. After being stirred at 25°C for 24 h, free JB was removed by 2 kD dialysis membrane at ambient temperature. The samples after dialysis were employed to determine the concentration of JB, and the samples after being dialysed were filtered 30 times with a filter under a pore diameter of 200 nm. After the solution was collected and centrifugated at (10,000 rpm × 2 min), the precipitates were washed with ddH_2_O 3 times to synthesize BPQDs@JB. The encapsulation efficiency (EE) and load efficiency (LE) of JB were calculated (Zhao et al., 2020) using EE% = Total mass of JB- mass of JB in supernatant/Total mass of JB × 100%; LE% = Total mass of JB-total mass of JB in supernatant/(Total mass of JB-total mass of JB in supernatant) + mass of BPQDs × 100%.

### Characterization of BPQDs@JB

The morphology of BPQD was detected with a transmission electron microscope (TEM, Tecnai G2 F20, USA). The particle size and zeta potential values of BPQD were respectively determined with a Zetasizer Nano ZS (Malvern Nano series, Malvern, UK). The absorbance of BPQDs, JB, and BPQDs@JB was measured by UV/Vis spectroscopy (ScanDrop, Analytik Jena, Germany).

### BPQDs@JB Release Properties for JB *in vitro*

The *in vitro* drug release experiments were carried out under pH 7.4 and pH 5.0 conditions to determine the ability of BPQDs@JB to release JB in a pH-dependent manner. Thereafter, 1 mL of BPQDs@JB was added to 20 mL of PBS solutions with pH values of 7.4 and 5.0, respectively, and dialyzed at 37°C. The absorbance of JB in the dialysate was measured by a microplate reader (PerkinElmer EnSpire, USA) (Shang et al., 2019). The concentration and cumulative release of JB were calculated complying with the standard curve. The cumulative release percentage (%) of JB in BPQDs@JB at each time point under different conditions was calculated, and the cumulative release curve of time drug was plotted (Zhou et al., 2017).

### Cell Viability of BPQDs@JB Assessed by CCK-8

After Raji cells were inoculated into 96-well plates with 5 × 10^3^ cells per well for 24 h, they were treated with 0, 5, 10, 20, 40, and 80 μmol/L of the free JB drug. After 24 h of incubation, 10 μL of CCK-8 was added to each well for an additional 4 h of incubation. Absorbance was then detected at 450 nm. Raji cells were also treated with PBS, BPQDs, JB, and BPQDs@JB, according to the above steps, and the cell survival rates were respectively detected.

### Apoptosis Assay of BPQDs@JB by Flow Cytometry *in vitro*

To evaluate the antitumor effect of BPQDs@JB *in vitro*, we employed an Annexin V-FITC/PI apoptosis kit and detected the apoptosis of Raji cells. Briefly, Raji cells were inoculated into a 6-well plate with 1 × 10^5^ cells/well. Thereafter, the cells were treated with PBS, BPQDs, JB, and BPQDs@JB for 24 h. The level of apoptosis was detected by flow cytometry (FACS CantoTM II, BD, USA).

### ROS Assay of BPQDs@JB by Flow Cytometry *in vitro*

Raji cells in logarithmic growth phase were inoculated in 6-well plates at the density of 1 × 10^5^ cells/mL. After being cultured for 12 h, the cells were administrated with PBS, BPQDs, JB and BPQDs@JB, respectively. After being cultured for 24 h, the cells were collected. DCFH-DA was diluted to 10 μmol/L in final concentration with serum-free RPMI-1,640 medium. Each well was incubated with 100 μL diluted DCFH-DA, at 37°C, 5% CO_2_ incubator for 20 min. The cells were washed gently with serum-free RPMI-1,640 medium 3 times to remove the DCFH-DA that did not enter the cells (the cells should not be sucked out). Photographs were taken under an inverted fluorescence microscope. Flow cytometry was adopted to detect the fluorescence intensity before and after the action of the drug. The excitation wavelength was 488 nm, and the emission wavelength was 525 nm (Li et al., 2019).

### Establishment of the Tumor-Bearing Mouse Model of Lymphoma

Six-week-old Balb/c nude mice were purchased from Hunan SJA Experimental Animal Co., Ltd (China). Each mouse was subcutaneously injected 6 × 10^7^ Raji cells/100 μL. When a tumor volume of 100 mm^3^ was achieved, this indicated the successful establishment of the tumor model.

### BPQDs@JB Treatment in Lymphoma-Bearing Mice

After animals were randomly divided into four groups (*n* = 3), 100 μL of PBS, BPQDs, JB, and BPQDs-JB was injected into the tail vein of mice every 3 days for a total of four times. Thereafter, the tumor size of animals were measured every 3 days. On day 21, the animals were killed and their tumors and visceral tissues (heart, liver, spleen, lung, and kidney) were collected. Tumor tissues and organs were fixed with 4% formalin and frozen at −80°C. The fixed tissues were embedded in paraffin and sliced into sections for H&E staining, immunofluorescent staining, and immunohistochemical staining.

### ROS and TUNEL Assays *in vivo*

Briefly, paraffin-embedded tissue samples were dewaxed for antigen recovery. Thereafter, the nuclei of apoptotic cells were identified with a TDT *in situ* apoptosis kit. The morphology of cells was observed and images were captured with a light microscope. ROS was observed via DCFH-DA immunofluorescence staining. The nucleus was stained with DAPI. The images were analyzed and captured with a laser confocal microscope (LCFM, LSM700, Germany).

### Statistical Analysis

Data were assessed by SPSS 18.0 and expressed as mean ± SD. Intergroup differences were assessed by One-Way ANOVA, followed by Tukey's *post-hoc* test (^*^p < 0.05, ^**^p < 0.01, ^***^p < 0.001, and ^****^p < 0.0001).

## Results and Discussion

### Preparation and Characterization of BPQDs@JB Nano-TCM

**Figure 2:**
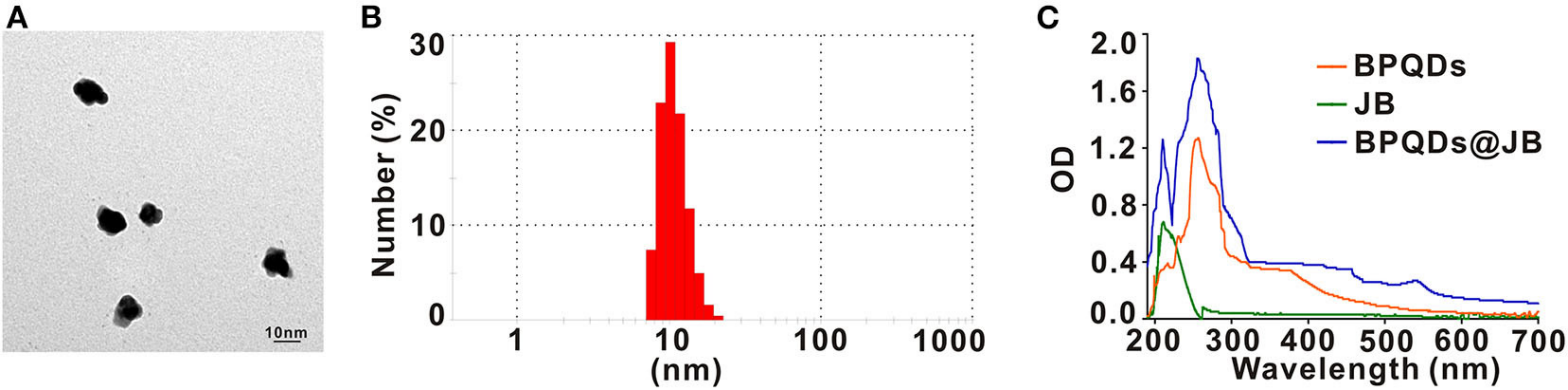
Characterization of BPQDs@JB. **(A)** TEM images of BPQDs. Scale bar: 10 nm. **(B)** Hydraulic radius of BPQDs. **(C)** UV/Vis spectra of BPQDs, JB, and BPQDs@JB.

To prepare the BPQDs@JB nano-TCM, JB was first loaded into BPQDs to derive BPQDs@JB (Figure 1). Through TEM imaging, BPQDs and BPQDs@JB were recognized to be monodispersed (Figure 2A and Figure S1A), the obtained BPQDs and BPQDs@JB were 12 nm in size on average by dynamic light scattering (Figure 2B and Figure S1B). Since BPQDs is negatively charged in water (Tayari et al., 2015). Small molecule drugs with positive charge are likely to be adsorbed by BPQDs by electrostatic interaction (Chen et al., 2017). Thus, the interaction mechanism between BPQDs and JB may be the electrostatic interaction of charge adsorption. As shown in Figure S1C, the Zeta potentials of BPQDs and BPQDs@JB were −37.26 ± 1.7 mV, −28.07 ± 1.6 mV, respectively. UV/Vis spectra (Figure 2C) of BPQDs@JB revealed absorption peaks at 256 and 211 nm, which align with the absorption peaks of BPQDs and JB, respectively. Therefore, BPQDs@JB was successfully assembled.

### EE and LE of the Drug, and the Release Rate of BPQDs@JB

**Figure 3:**
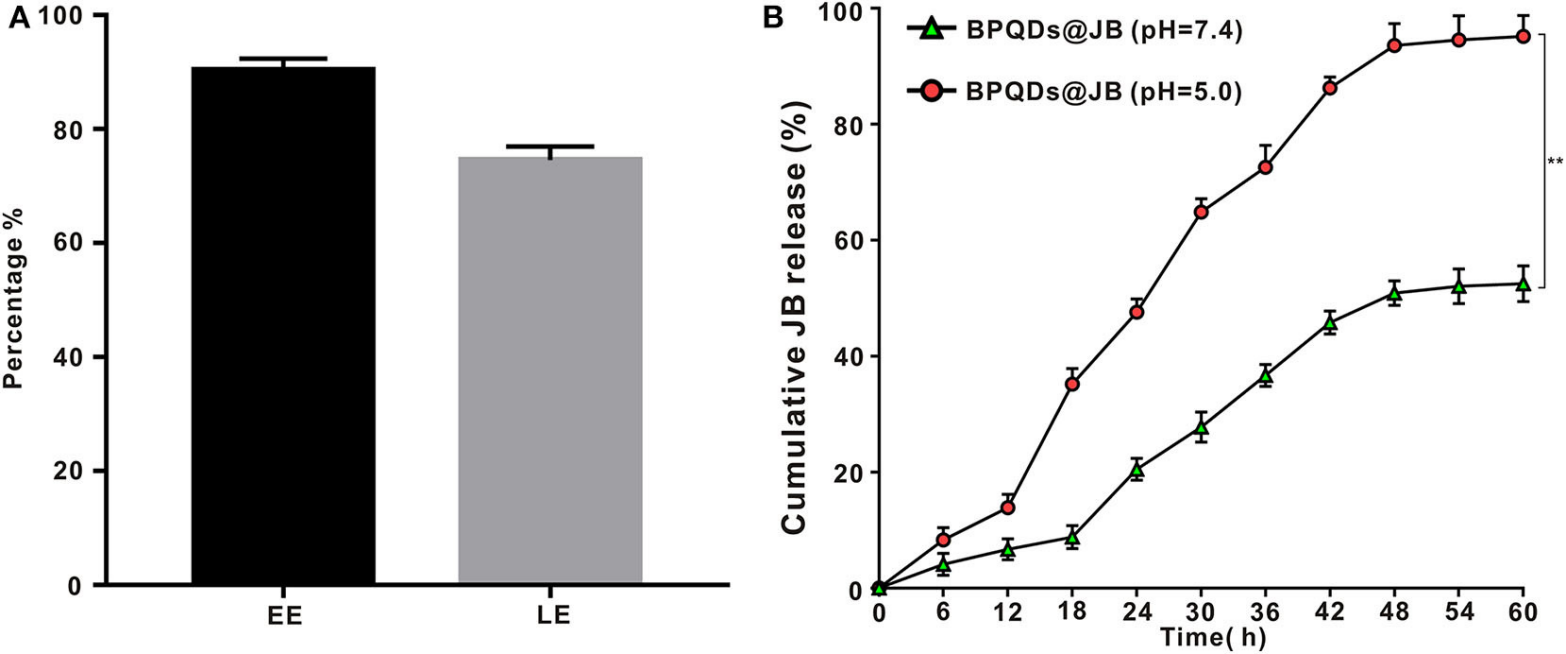
Drug LE of BPQDs and the release rate of BPQDs@JB. **(A)** EE and LE of BPQDs. **(B)** Cumulative release rates of JB from BPQDs@JB at different pH-values (7.4 and 5.0). Compared with the BPQDs@JB (pH = 7.4) group, **p < 0.01.

As new two-dimensional material, black phosphorus, which has good biodegradability, could be utilized in drug delivery (Chen et al., 2017). Compared to BP nanosheets, BPQD is more attractive for drug delivery systems owing to its smaller size (Geng et al., 2018). By using BPQD-loaded drugs, we found that the EE and LE of JB in the BPQDs@JB nano-TCM were 90.3 ± 2.1% and 74.6 ± 2.4%, respectively (Figure 3A). Thereafter, we proceeded to evaluate the drug release characteristics of BPQDs@JB. As shown in Figure 3B, when BPQDs@JB exhibited a pH of 5.0, the cumulative release rates of JB at 6, 12, 18, 24, 30, 36, 42, 48, 54, and 60 h were 8.3 ± 2.1%, 13.9 ± 2.3%, 35.2 ± 2.7%, 47.6 ± 2.3%, 64.9 ± 2.3%, 72.6 ± 3.8%, 86.3 ± 1.9%, 93.6 ± 3.8%, 94.5 ± 4.2%, 95.2 ± 3.6%, respectively, and at pH 7.4, the cumulative release rate of JB at 6, 12, 18, 24, 30, 36, 42, 48, 54, and 60 h were 3.9 ± 1.3%, 5.9 ± 1.8%, 8.8 ± 2.0%, 20.5 ± 1.9%, 27.8 ± 2.6%, 36.5 ± 1.9%, 45.3 ± 2.0%, 50.9 ± 2.1%, 52.1 ± 3.0%, 52.5 ± 3.1%, respectively. Therefore, the release rate of JB at pH 5.0 was higher than that at pH 7.4. Moreover, an increase in cumulative drug release was identified, thereby indicating the accelerated degradation of BPQDs under acidic conditions (Zhou et al., 2018). Because the tumor environment is weakly acidic (Matsumoto et al., 2017), BPQDs@JB is a type of nano-TCM released in response to pH, which is beneficial for the treatment of tumor. Generally, the above findings demonstrate that BPQD is an efficient drug carrier, and an acidic environment enables the release of JB from BPQDs@JB.

### Effect of BPQDs@JB on Raji Cell Viability *in vitro*

**Figure 4:**
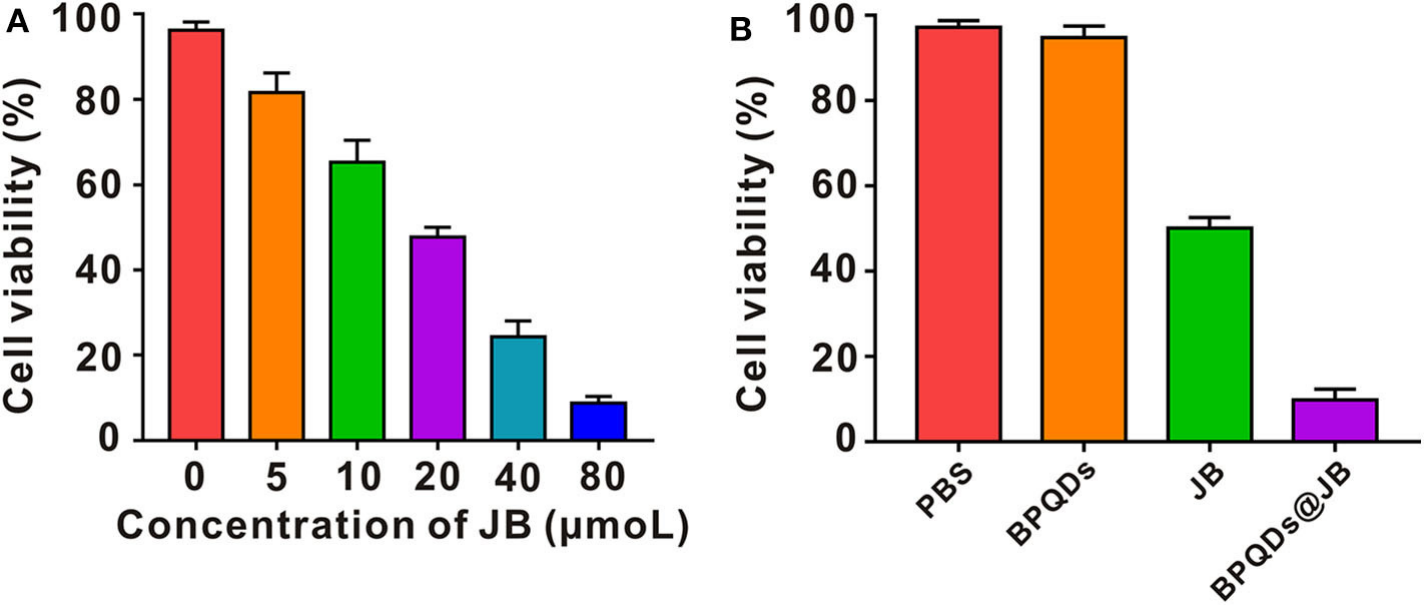
Effect of BPQDs@JB on Raji cell viability *in vitro*. **(A)** Viability of Raji cells following treatment with different concentrations of JB (IC50). **(B)** Raji cell viability following the administration of PBS, BPQDs, JB, and BPQDs@JB for 24 h. Data are presented as mean ± SD (*n* = 3).

Raji cells were isolated and established from Burkitt's lymphoma of the left upper jaw in an 11-year-old black boy, i.e., the origin of B cells (Theofilopoulos et al., 1974). Since Burkitt's Lymphoma is considered a highly invasive and malignant non-Hodgkin's Lymphoma (Ribrag et al., 2016), Raji cells were taken to conduct the experimental study. Raji cells were administrated with BPDs at concentrations of 0, 0.25, 0.5, 1.0, and 2.0 mg/mL for 24 h, respectively, and the cell viability rate was determined by CCK-8. As shown in supporting Information Figure S2, the cell viability rate of Raji cells incubated with a series of concentrations of BPDs for 24 h, of which the viability rate of Raji cells administrated with 2.0 mg/mL CCM@MSNs was as high as 90%. Therefore, BPQDs (concentration of 0.25 mg/ml) were taken as the follow-up experiment. Then, Raji cells were treated with different concentrations of JB. According to the cell survival rate, the IC50 value of JB was ~20 μmol/L (Figure 4A). Thus, JB (concentration of 20 μmol/L) for the follow-up experiment. To compare the survival rate of Raji cells between BPQDs@JB and free JB, the JB in BPQDs@JB was diluted to 20 μmol/L in concentration for the follow-up experiment. Thereafter, Raji cells were respectively treated with PBS, BPQDs, JB, and BPQDs@JB. As shown in Figure 4B and Table 1, the CCK-8 results revealed that PBS and BPQDs had no evident toxic effects on Raji cells. The cell viability of Raji cells administrated with free JB was 50.08 ± 2.37%. However, the cell viability of Raji cells administrated with BPQDs@JB was (19.85 ± 1.02%). Compared with JB group, the cell viability of BPQDs@JB group decreased significantly. Therefore, BPQDs@JB nano-TCM has a stronger anti-lymphoma effect than using JB alone.

**Table 1:** A table to summarize all data in Figure 4B by putting the respective numbers.

	**PBS**	**BPQDs**	**JB**	**BPQDs@JB**
Cell viability (%)	97.17 ± 1.68	94.83 ± 2.56	50.08 ± 2.37***	19.85 ± 1.02****

Compared with the PBS group, ***p < 0.001, ****p < 0.0001.

### ROS and Apoptosis Assessment by Flow Cytometry *in vitro*

**Figure 5:**
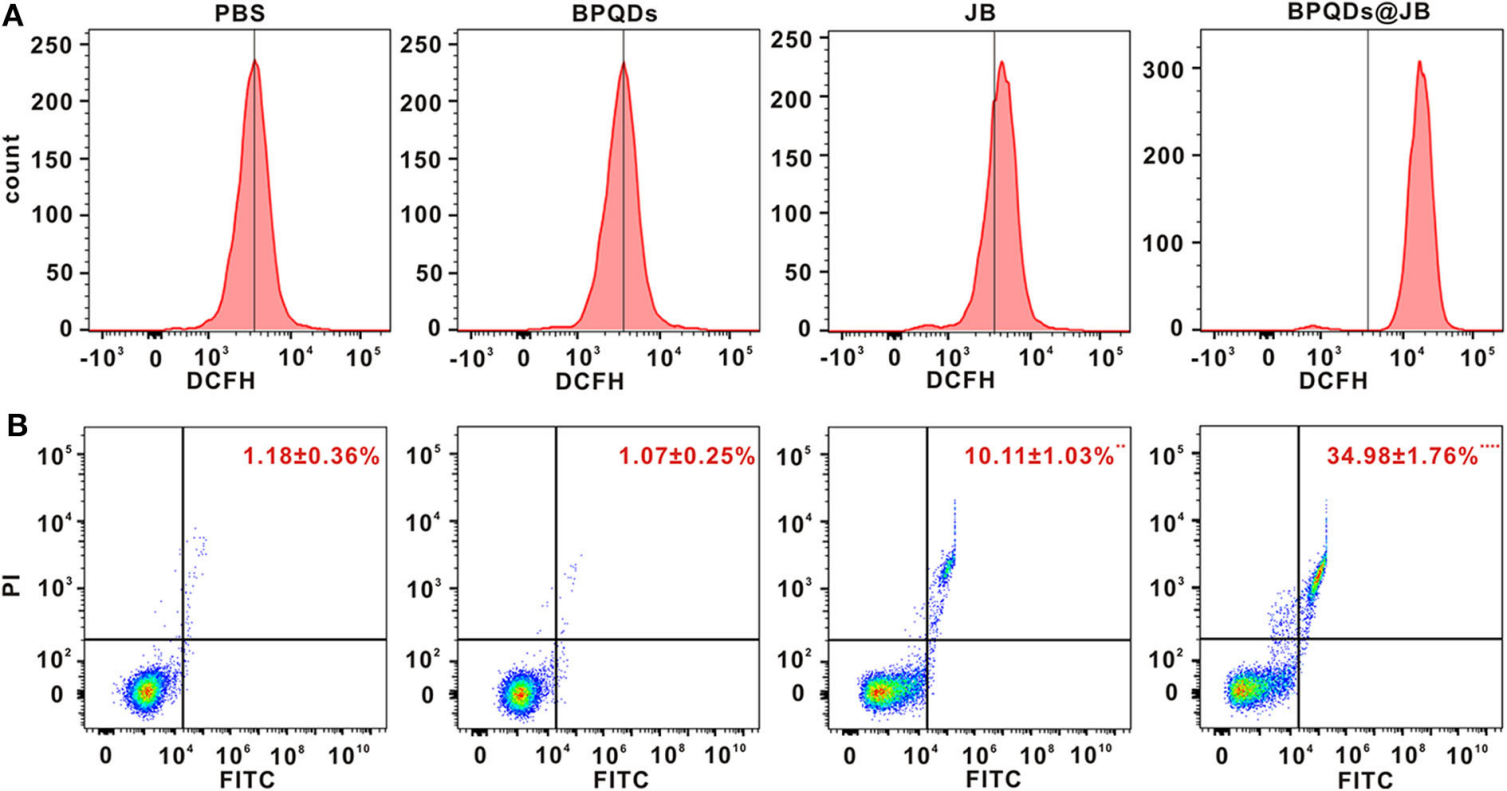
ROS and Apoptosis assessment by flow cytometry *in vitro*. **(A)** Flow cytometric detection of ROS level in Raji cells treated with PBS, BPQDs, JB, and BPQDS@JB for 24 h. **(B)** Flow cytometric assessment of the level of apoptosis in Raji cells administered PBS, BPQDs, JB, and BPQDS@JB for 24 h. Data are presented as mean ± SD (*n* = 3). Compared with the PBS group, **p < 0.01, ****p < 0.0001.

Oxidative stress caused by reactive oxygen species (ROS) might serve as an important factor in tumor occurrence and development. Recent studies have shown that excessive ROS can lead to the apoptosis and necrosis of tumor cells (Wu et al., 2019). Ren et al. (2016) revealed that psoralen induces DNA damage and apoptosis in breast cancer cells by inducing ROS production. To verify whether JB can kill Raji cells via ROS production, we sought to determine the effect of PBS, BPQDs, JB, and BPQDs@JB on Raji cells by flow cytometry. As shown in Figure 5A and Figure S3A, compared to treatment with PBS and BPQDs, treatment with the BPQDs@JB nano-TCM or JB could cause a shift to the right in the histogram, suggesting that the latter two groups can produce a large amount of ROS, with BPQDS@JB producing more ROS than JB. The same result is shown in Figure S3B, the red fluorescence intensity of BPQDs@JB was significantly stronger than that of other groups, revealing that BPQDs@JB can significantly increase ROS in Raji cells. Flow cytometry was subsequently employed to further detect the apoptotic effect of PBS, BPQDs, JB, and BPQDs@JB on Raji cells. As shown in Figure 5B, compared to the PBS group, the BPQD group did not cause significant apoptosis of Raji cells. However, after treatment with BPQDs@JB nano-TCM, the apoptotic rate of Raji cells was 34.98 ± 1.76%, a value higher than that achieved following treatment with JB (10.11 ± 1.03%). This finding indicates that BPQDs@JB nano-TCM could better induce Raji cell apoptosis than free JB. Therefore, BPQDs@JB nano-TCM can induce apoptosis of Raji cells via ROS production.

### Antitumor Effects of BPQDs@JB *in vivo*

**Figure 6:**
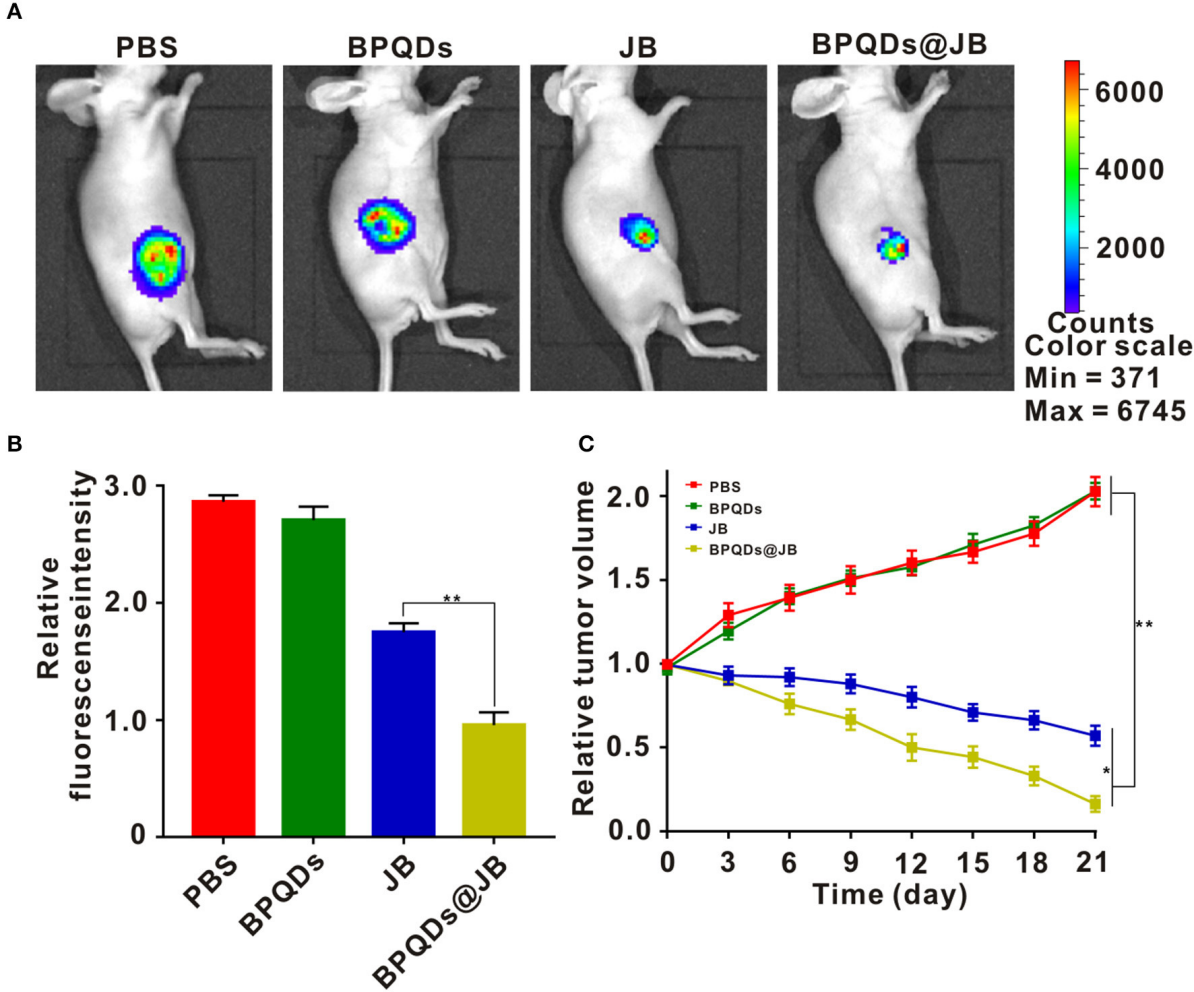
Antitumor effects of BPQDs@JB *in vivo*. **(A)** After intravenous injection of PBS, BPQDS, JB, and BPQDs@JB into the tail vein of nude mice, the fluorescence signal images of tumor tissues were detected on day 21. **(B)** Semi-quantitative evaluation of the fluorescence signal of tumor tissue samples in each group. **(C)** Changes in tumor volume in the Raji tumor-bearing mouse model during treatment. Data are expressed as mean ± SD (*n* = 3; *p < 0.05, **p < 0.01).

Herein, we employed the Raji tumor-bearing mouse model to elucidate the anti-lymphoma effects of BPQDs@JB *in vivo*. PBS, BPQDs, JB, and BPQDs@JB were injected into the tail vein of Raji tumor-bearing mice. On day 21, these nude mice were observed with a live imager. As shown in Figures 6A,B, the tumor fluorescence signal intensity of the JB group and BPQDs@JB group was significantly weaker than that of the PBS group and BPQDs group. Further, the tumor fluorescence signal intensity of the BPQDs@JB group was weaker than that of the other groups. Similarly, as illustrated in Figure 6C, after 21 days of monitoring the changes in tumor volume, BPQDs@JB exerted a better anti-lymphoma effect than JB alone. These findings suggest that BPQDs@JB exhibits an enhanced antitumor effect *in vivo*.

### Change in ROS Level and the Results of the TUNEL Assay

**Figure 7:**
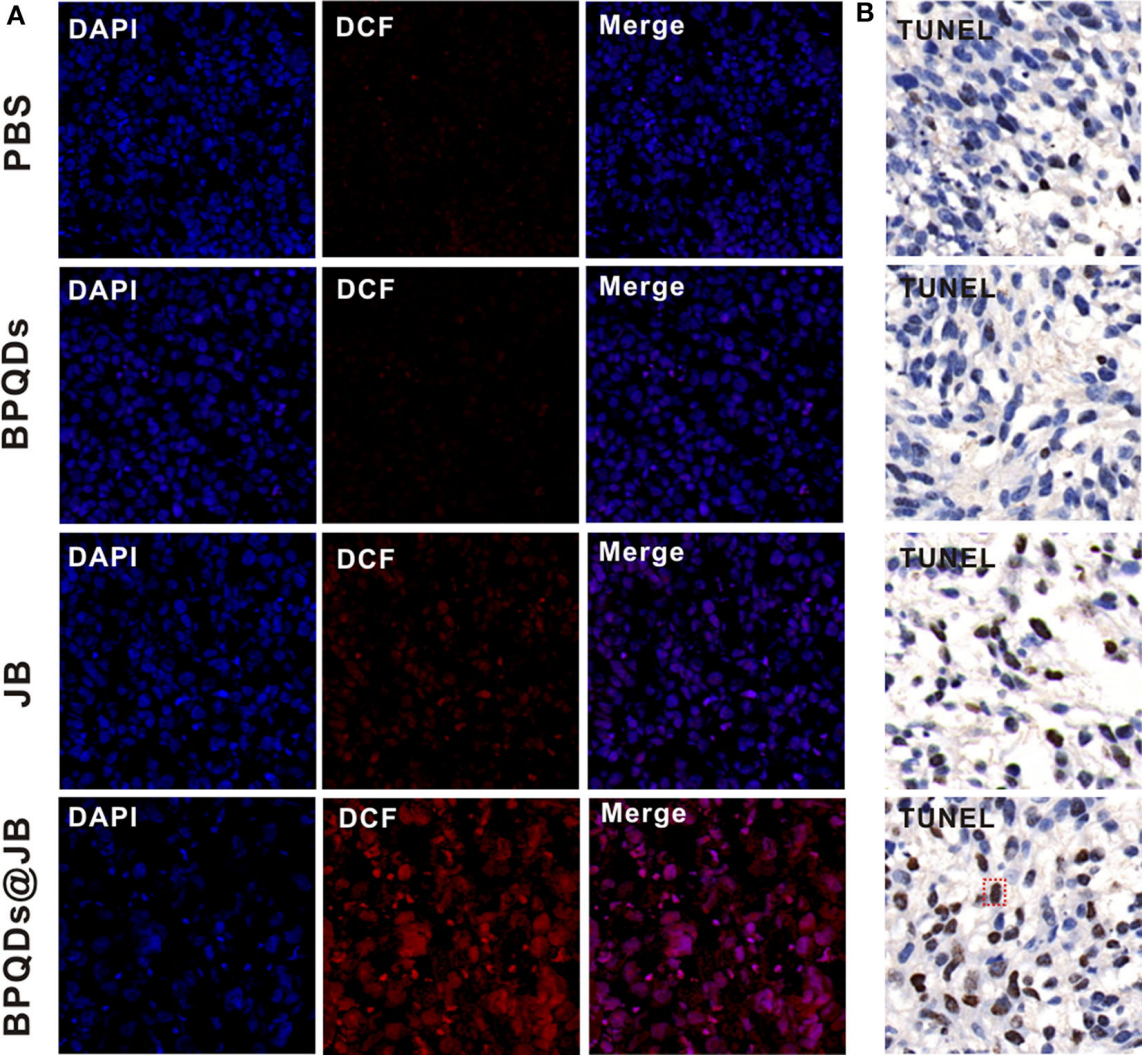
Changes in the level of ROS and the results of the TUNEL assay. **(A)** After 21 days of administering PBS, BPQDs, JB, and BPQDs@JB into the tail vein of mice, the level of ROS in the tumor tissue was detected by immunofluorescence. **(B)** Tumor tissues were assessed at 21 days after intravenous injection of PBS, BPQDs, JB, and BPQDs@JB via TUNEL assays.

imbalance in ROS level in tumor cells can activate the apoptotic pathway and induce apoptosis (Uthaman et al., 2019). Therefore, breaking the redox state in tumor cells is an effective strategy for the treatment of tumors. Presently, this strategy has been employed to synthesize numerous drugs, which are either being developed or have entered clinical trials, and exhibit good anticancer effects (Martin-Cordero et al., 2012; Raza et al., 2017). As BPQDs@JB nano-TCM produced a large amount of ROS against lymphoma *in vitro*, we opted to further detect the level of ROS produced by BPQDs@JB *in vivo*. As shown in Figure 7A, the tumor tissue sections treated with BPQDS@JB exhibited a Conspicuous red fluorescence. However, the fluorescence exhibited by sections treated with JB was significantly weaker than that exhibited by sections treated with BPQDS@JB. A slight red fluorescence was also observed in the PBS group and BPQD group, suggesting that most ROS production occurred in the BPQDS@JB group. We proceeded to use the TUNEL method to detect the level of apoptosis in tumor tissue. As shown in Figure 7B, the number of positive (brown) nuclei in the BPQDs@JB group was significantly greater than that in other groups. Such finding indicates that the results of TUNEL detection in tumor tissue sections *in vivo* were consistent with those of the apoptosis induced by BPQDs@JB *in vitro*. These results suggest that BPQDs@JB can produce excessive ROS to inhibit tumor and interfere with the new strategy of the redox state against lymphoma.

### Safety Evaluation of Important Organs by BPQDs@JB

**Figure 8:**
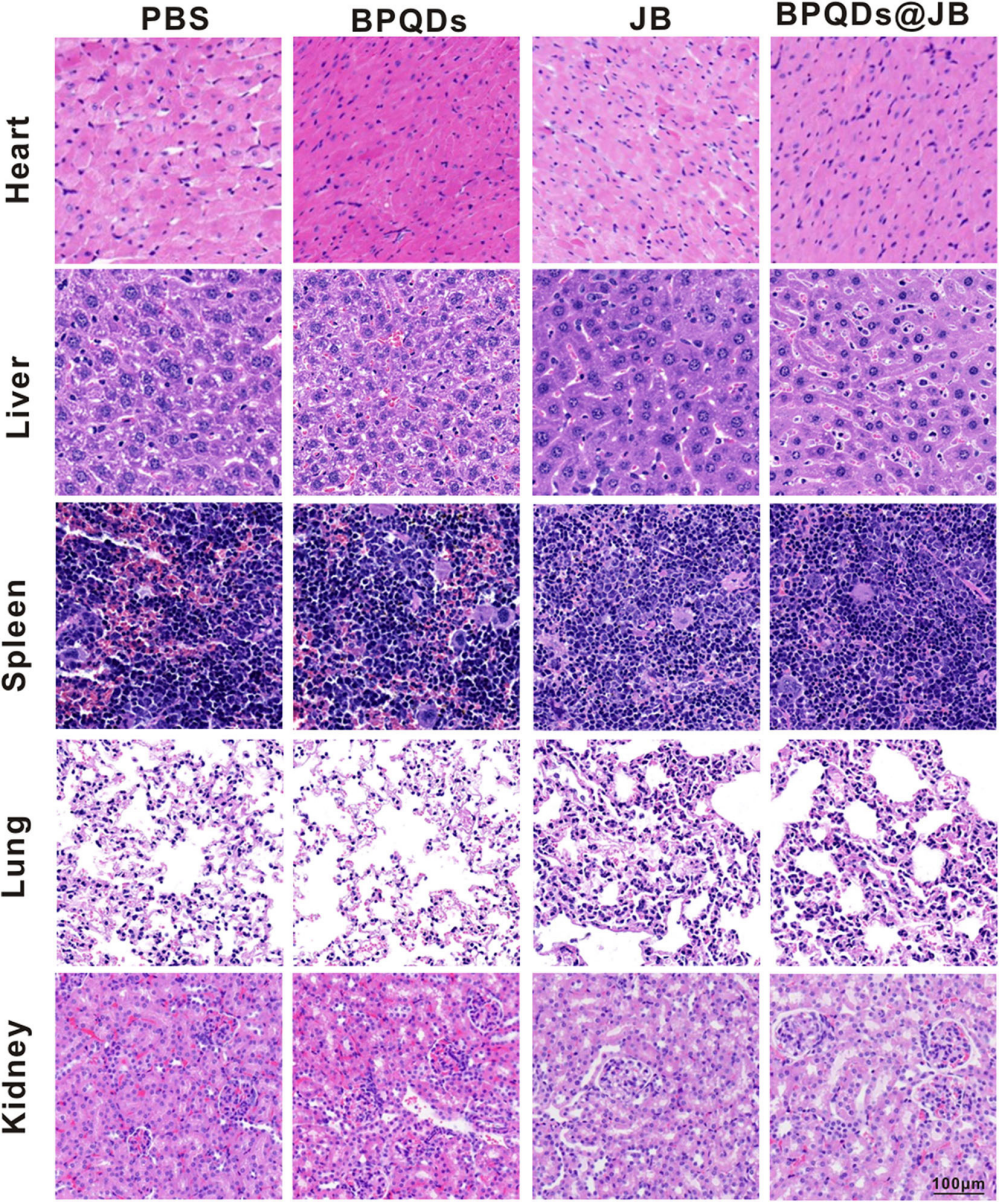
PBS, BPQDs, JB, and BPQDS@JB were injected into the tail vein of nude mice for 21 days. The heart, liver, spleen, lung, and kidney of nude mice were retrieved for histological imaging. Scale bar: 100 μm.

BPQD nano-TCM exerted a significant anti-lymphoma effect *in vitro* and *in vivo*. To verify the safety of BPQD nano-TCM *in vivo*, we used H&E staining to determine its toxic effect on the heart, liver, spleen, lung, and kidney. The histological data of the heart, liver, spleen, lung, and kidney revealed no abnormalities in the PBS group, BPQD group, JB group, and BPQDs@JB group (Figure 8). There were no abnormal changes in WBC, HGB, PLT, ALT, AST, BUN, CRE, CK, and Myo in nude mice treated with PBS, BPQDs, JB, and BPQDs@JB (Supporting Table 1). Such findings demonstrate that BPQDs@JB did not cause any side effects and may serve as a safe and effective nano-TCM.

## Conclusions

In the present study, we revealed that the newly derived two-dimensional material, BPQD, exhibit many properties with a high drug-loading rate, which is similar to an aircraft carrier, thereby enabling its transport of numerous drug molecules. The release of JB from BPQDs@JB can be accelerated in the acidic microenvironment of the tumor. As a result, BPQDs@JB nano-TCM kills lymphoma cells by regulating ROS. Because of these characteristics, BPQD is a non-toxic and efficient drug delivery platform. To summarize, herein, we revealed the role and potential mechanism of BPQDs@JB nano-TCM in the treatment of lymphoma. Hopefully, it can provide a novel idea for the treatment of lymphoma.

## Data Availability Statement

All datasets generated for this study are included in the article/Supplementary Material.

## Ethics Statement

The animal study was reviewed and approved by the experimental protocols involving all mouse in the present study were approved by the Institutional Animal Care and Use Committee of Xiangya Medical College of Central South University, and the mouse were kept according to the institutional ethical guidelines of Central South University.

## Author Contributions

RG and XN designed the experiment. QZ carried out the experiment. JL, BW, YS, XH, HD, and HL contributed to analyze the experimental results. QZ wrote the manuscript. All authors contributed to the article and approved the submitted version.

## Conflict of Interest

The authors declare that the research was conducted in the absence of any commercial or financial relationships that could be construed as a potential conflict of interest.
