# Supplementary material for: A Nano-Traditional Chinese Medicine Against Lymphoma That Regulates the Level of Reactive Oxygen Species
Source: Front Chem. 2020 Jul 14;8:565. doi: 10.3389/fchem.2020.00565 (PMC7381219; doi:10.3389/fchem.2020.00565)
Supplement: Supplementary file 1 [file Data_Sheet_1.PDF]

Supporting Information

A Nano-traditional Chinese medicine against Lymphoma that regulates the level of reactive oxygen species

Qiangqiang Zhao[1,2], Jian Li[1], Bin Wu[3], Yinghui Shang[1], Xueyuan Huang[1], Hang Dong[1], Haiting Liu[1], Rong Gui[1]* and Xinmin Nie[1]*

[1]Department of Blood Transfusion, the Third Xiangya Hospital, Central South University, Changsha 410013, P. R. China

[2]Department of Hematology, the Qinghai Provincial People's Hospital, Xining 810007, P. R. China

[3]Department of Transfusion Medicine, Wuhan Hospital of Traditional Chinese and Western Medicine, Tongji Medical College, Huazhong University of Science and Technology, Wuhan 430022, P. R. China,

*Corresponding author. Phone/Fax: +86-731-8861 8513

E-mail address: zndxgr@163.com (R. Gui)

*Corresponding author. Phone/Fax: +86-731-8861 8520

E-mail address: niexinmin7440@sina.com (X. m. Nie)

Table of Contents

1. Additional Experimental Section 3
    1.1. The particle sizes and zeta potential values of BPQDs@JB. 3
    1.2. Study on biosafety of BPQDs@JB *In Vivo*. 3
2. Additional Results 4
3. References: 8

1. Additional Experimental Section

1.1. The particle sizes and zeta potential values of BPQDs@JB.

The BPQDs@JB with organic solvent N-methyl-2-pyrrolidone (NMP) (99.5%, anhydrous)(Shao et al., 2016), and determined the particle size and Z potential of BPQDs@JB with a Zetasizer Nano ZS (Malvern Nano series, Malvern, UK).

1.2. Study on biosafety of BPQDs@JB *In Vivo*.

Blood samples were collected from mice for hematological and biochemical analysis. The whole blood was collected for anticoagulation in a test tube equipped with EDTA, and the blood cell count (WBC, HGB and PLT), was detected using the blood cell analyzer (BC-5390, Mindray, China). The serum enzyme level (Liver function index: ALT, AST; Renal function index: BUN, CRE; and Cardiac function index: CK, Myo) were determined on the 7100 automatic biochemical analyzer (Hitachi, Japan), respectively.

2. Additional Results

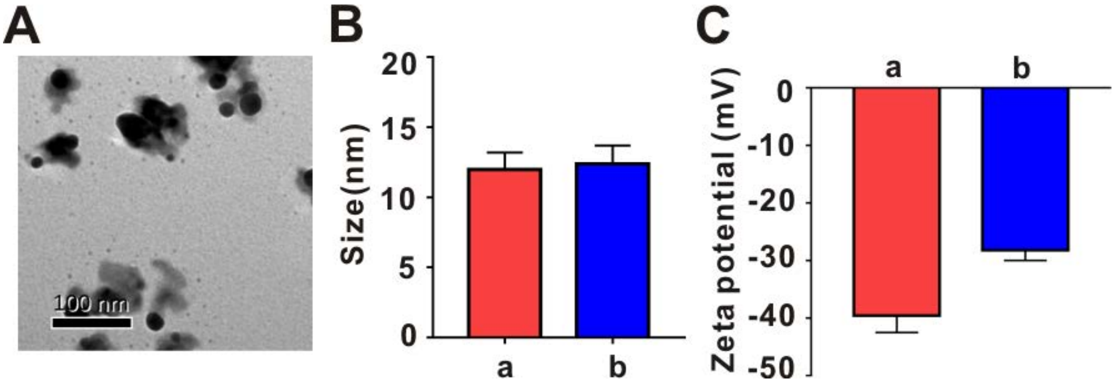

Figure S1. (A) TEM images of BPQDs. Scale bar: 100 nm. The particle sizes and (B) zeta potential values (C) of BPQDs, BPQDs@JB. a) BPQDs, b) BPQDs@JB. Data are mean ± SD (n = 3).

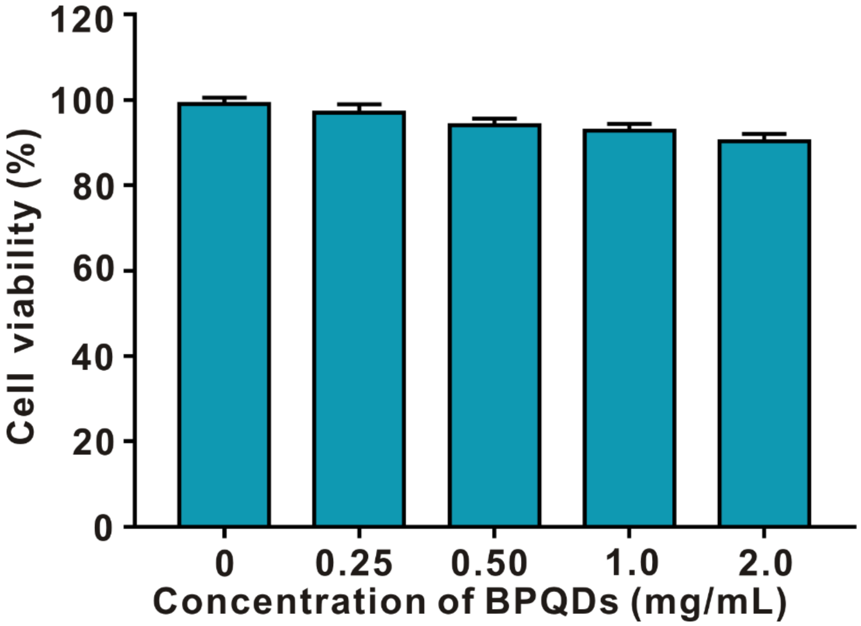

Figure S2. Cell viability rate of Raji cells upon treatment with different concentration of BPQDs. Data are expressed as mean ±SD (n = 3).

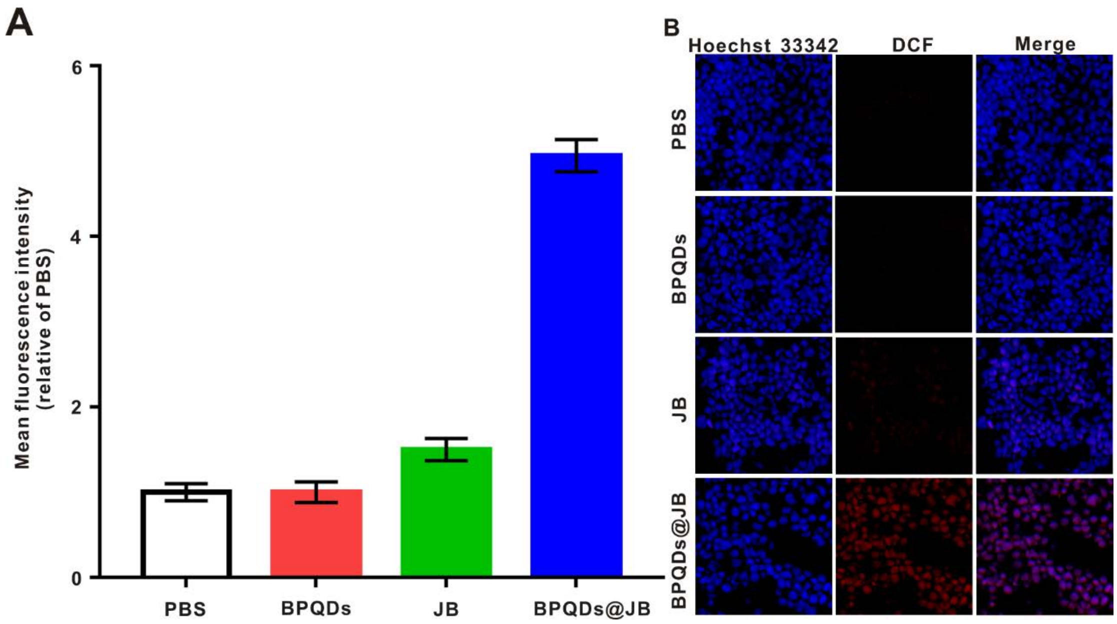

Figure S3. (A) The average fluorescence intensity of PBS, BPQDs, JB and BPQDs@JB (relative of PBS) was semi-quantitatively analyzed. (B) Raji cells were administrated with PBS, BPQDs, JB and BPQDs@JB for 24 h, and then photographed and imaged under an inverted fluorescence microscope. Data are presented as mean ± SD (n = 3).

	PBS	BPQDs	JB	BPQDs@JB
WBC(10^9/L)	8.02±0.97	7.96±0.83	8.17±0.10	7.84±0.72
HGB(g/dL)	14.52±1.62	14.75±1.36	14.61±1.19	14.64±1.46
PLT(10^{11}/L)	13.51±2.16	13.28±1.94	13.49±2.01	13.62±2.21
ALT(U/L)	53.29±4.37	52.61±3.75	54.37±4.26	52.96±3.89
AST(U/L)	126.43±19.65	119.71±17.52	122.67±18.21	124.83±19.02
BUN(mmol/L)	8.62±1.08	8.76±1.25	8.59±1.01	8.70±1.14
CRE(μmol/L)	85.67±22.36	88.32±24.82	86.51±21.69	87.42±23.06
CK(U/L)	678.5±131.4	694.3±146.6	680.2±129.5	674.7±135.0
Myo(ng/mL)	81.2±5.7	85.7±4.9	88.2±6.1	82.6±4.2

Supporting Table 1. Hematological index (WBC, HGB and PLT), Liver function index (ALT, AST), Renal function index (BUN, CRE), and Cardiac function index (CK, Myo) were detected at 21 day after intravenous injection of PBS, BPQDs, JB and BPQDs@JB, respectively. Data are mean ± SD (n = 3).

3. References:

Shao, J., Xie, H., Huang, H., Li, Z., Sun, Z., Xu, Y., Chu, P. K. (2016). Biodegradable black phosphorus-based nanospheres for in vivo photothermal cancer therapy. *Nat Commun, 7*, 12967. doi: 10.1038/ncomms12967
